# Mainstreaming One Health

**DOI:** 10.1007/s10393-012-0772-8

**Published:** 2012-07-10

**Authors:** Jakob Zinsstag, John S. Mackenzie, Martyn Jeggo, David L. Heymann, Jonathan A. Patz, Peter Daszak

**Affiliations:** 1 Department of Epidemiology and Public Health, Swiss Tropical and Public Health Institute, 4002 Basel, Switzerland; 2 University of Basel, Petersplatz 1, 4003 Basel, Switzerland; 3 Faculty of Health Sciences, Curtin University, P.O. Box U1987, Perth, WA 6845 Australia; 4 The Burnet Institute, Melbourne, VIC 3004 Australia; 5 Australian Animal Health Laboratory, P.O. Box 100, Geelong, VIC Australia; 6 Centre on Global Health Security, Chatham House, London, UK; 7 Infectious Disease Epidemiology, London School of Hygiene and Tropical Medicine, London, UK; 8 Global Health Institute, University of Wisconsin, 1710 University Avenue, Madison, WI 53726 USA; 9 EcoHealth Alliance, 460 West 34th Street, 17th Floor, New York, NY 10001 USA

The term ‘One Medicine’ was coined by Schwabe (1984) and focuses attention on the commonality of human and animal health. The underlying concept is traceable to the late nineteenth century, in contributions of the German pathologist and architect of social medicine Rudolf Virchow (Saunders 2000; Zinsstag and Weiss 2001). Schwabe states that there is no difference in paradigm between human and veterinary medicine and that both medicines have the same scientific foundations. Yet, human and animal health developed during the nineteenth and twentieth centuries into fairly segregated disciplines or ‘silos’, separated at the academic, governance and application levels. In recent decades, the concept of ‘One Medicine’ evolving to ‘One Health’ has gained momentum worldwide after the SARS outbreak in 2003, and then driven by fears of a possible pandemic of H5N1 avian influenza (Zinsstag et al. 2005; Worldbank 2010). One Health now encompasses a broad agenda from zoonotic infections (Roth et al. 2003), food safety, to health services delivery (Schelling et al. 2005), and beyond.

Consideration of ‘One health’ calls for a reflection on the human–animal relationship from its natural history to cultural influences. Molecular genetics suggests that the human genome is 99 % similar to great apes and 95 % to pigs. Genetically, humans can be seen as only slightly remodelled chimpanzee-like apes (Wildman et al. 2003). From a biological perspective, humans should consider such domesticated animals and wildlife as close relatives, with similar capacity to transmit infectious organisms to us as members of our human family. We should therefore treat our relationship with other animal species as part of a continuum across which pathogens can emerge and spread, exploiting new niches as we change our interactions, and moving into and out of erstwhile distinct species, regions or communities (Daszak et al. 2000).

Nevertheless, the contemporary human–animal relationship is complex and profound, ranging from exploitation of livestock for food and anthropomorphisation of animals as pets, to live ‘wet markets’ and international trade in animal species; these processes, which are highly culturally determined, create interfaces between animals and humans, which lead in some instances, to disease emergence. Additional driving mechanisms of potential disease emergence or resurgence stem from: indiscriminate destruction of native habitats for economic or agricultural development; biodiversity loss and niche invasions; induced genetic changes (e.g. antibiotic-resistant bacteria or pesticide-resistant mosquitoes); and environmental contamination (Patz et al. 2005).

Of these activities, probably the primary factor driving human and animal interaction is globalised livestock production, which tends to focus on maximising profit, sometimes with ethical implication. This leads to competing agendas as, ironically, moderate intensification of livestock production is the way out of poverty for millions of smallholder farmers. Similarly, naturalist movements sometimes claim that human rights extend to primates, whales or other species, yet these are not widely held beliefs. It is these dilemmas of aspiration for economic gain in a globalised economy, desire for social development and concern over animal welfare that largely determines the human–animal relationship. Intercultural work on the human–animal relationship requires a clarification of one’s own perspective in a self-reflective way. ‘What is my personal cultural/and ethical background that determines my relationship with animals and my concept of one health?’ Answers critically determine the emotional or financial value assigned to animals. Could this lead to a new subjectivism in Science? ‘One health’, for example, can be influenced by philosophical ramifications, that determine the method of economic analyses of the cost of infections that are transmissible between humans and animals (Narrod et al. 2012).

How can we benefit most from ‘One health’? Firstly, through the broad implications of closer cooperation between human and animal health sectors and recognising the linkages among humans, animals and the environment. This broad vision means that One Health solutions will benefit health, conservation and development. Secondly, mainstreaming a ‘One Health’ approach should lead to better health for humans and animals and financial savings to society from such a closer cooperation between the sectors *which could not be obtained if they worked in separation*. Recently, ‘One health’ conceptual thinking has evolved towards systemic approaches that consider health as an outcome of social–ecological systems. This includes concerns about social equity and the ‘integrity’ of the environment (Zinsstag et al. 2011). ‘One Health’ is clearly part of the broader consideration of ecology and health.

There is, however, a large unfinished agenda in the ‘mainstreaming’ of one health that requires enhanced cooperation and communication between human and animal health. There are obstacles, many of which are economic, to broad transdisciplinary acceptance of the benefits gained from a One Health approach. These range from understanding and mitigating the determinants of zoonoses and emerging infections to the prevention, detection and response when they occur in animal and/or human outbreaks. To overcome these obstacles, we urgently need stronger international leadership from the major international organizations—the World Health Organization (WHO), the Food and Agriculture Organization (FAO) and the World Animal Health Organization. The Office of the United Nations Secretary General’s Special Representative for Food Security and Nutrition may also play a crucial role. A tripartite agreement has been signed by WHO, FAO and OIE for sharing responsibilities and coordinating global activities to address health risks at the animal–human–ecosystems interface (Anonymous 2010). Stronger implementation of this agreement and associated advocacy is essential to give credibility and support to the One Health concept, and to ensure national One Health planning that can better respond to zoonoses and food safety.

Working together under this agreement, these three international organisations could provide the evidence and rational answers to questions such as: What are the direct benefits of joint human and animal communicable disease surveillance, along with environmental monitoring, for time to detection and response, the number of lives saved, and associated financial savings? How is power most effectively shared in leadership and chain of command that leads to effective and nimble implementation of integrated disease surveillance and control? What are potential benefits of joint antibiotic resistance surveillance, as in the Canadian Integrated Programme for Antimicrobial Resistance Surveillance (CIPARS)? What are benefits of joint laboratory facilities for human and animal communicable diseases, modelled after Canada’s National Microbiology Laboratory? What might be the benefits if cancer registries for humans and animals were joined (O’Brien et al. 2000)? How could the Performance of Veterinary Services (PVS) and the International Health Regulations (IHR), be linked in order to enhance their performance?

Answers to these and other such questions may not necessarily lead to new structures, new governance or even a ‘One Health’ society. Rather, the existing international organizations, by providing the scientific evidence and guidance based on this evidence, could provide what is needed to change current siloed practices that are our normal way of doing business. Let us continue to work in our disciplines and institutions to bring them closer by improved communication, greater collaboration and better information sharing. Fostering mutual respect amongst doctors and veterinarians and recognising and acknowledging the interdependence of health in humans and animals is a necessity. At the same time, new evidence is expanding on the dependence of both human and animal health on ecosystem functioning, generally termed as ‘ecosystem services.’ According to a 2011 US report of the President’s Council of Advisors on Science and Technology (PCAST), ‘Ecosystems and the biodiversity they embody constitute “environmental capital” on which human well-being heavily depends,’ (PCAST 2011).

However, two impediments will need to be overcome if One Health is to achieve its potential as a solution to global health issues that brings an *economy of disciplinary scale*. Firstly, the health of humans, livestock and wildlife are connected to, and often grounded in, the environment they inhabit. The importance of underlying environmental change to the spread of infectious agents across these populations is now widely appreciated. Indeed, understanding the ecology of diseases is often the way that solutions to outbreaks or disease emergence are formed. Therefore, One Health needs a far greater engagement of the ecological and environmental sciences to achieve its potential. Secondly, breaking down the siloed approach to health will not be possible unless funding from government agencies, ministries, and intergovernmental funding mechanisms supports new collaborations and new communication channels. This will be difficult because ministries often compete for funding. However, specific line item funding for inter-departmental, interagency and inter-institutional collaboration may provide a solution. They would almost certainly be incredible value for money, given the importance of zoonotic agents to global public health, livestock production and wildlife conservation. In summary, what can be achieved in one health will depend on the ability of society to understand and accept scientific evidence and guidance for one health. Operationalizing this guidance can be enhanced by understanding being gained from a growing body of social scientists working on these linkages. Mainstreaming ‘One health’ will lead to closer cooperation between human and animal health and with other health related sectors (i.e. social and environmental sciences and economics), and will provide a road map for developing a sustainable approach to diseases at the human–animal–ecosytems interface.
